# Mouse Senile Amyloid Fibrils Deposited in Skeletal Muscle Exhibit Amyloidosis-Enhancing Activity

**DOI:** 10.1371/journal.ppat.1000914

**Published:** 2010-05-20

**Authors:** Jinze Qian, Jingmin Yan, Fengxia Ge, Beiru Zhang, Xiaoying Fu, Hiroshi Tomozawa, Jinko Sawashita, Masayuki Mori, Keiichi Higuchi

**Affiliations:** 1 Department of Aging Biology, Institute on Aging and Adaptation, Shinshu University Graduate School of Medicine, Matsumoto, Japan; 2 Department of Pathology, Hebei Medical University, Shijiazhuang, China; 3 Division of Laboratory Animal Research, Research Center for Human and Environmental Science, Shinshu University, Matsumoto, Japan; Istituto Superiore di Sanità, Italy

## Abstract

Amyloidosis describes a group of protein folding diseases in which amyloid proteins are abnormally deposited in organs and/or tissues as fine fibrils. Mouse senile amyloidosis is a disorder in which apolipoprotein A-II (apoA-II) deposits as amyloid fibrils (AApoAII) and can be transmitted from one animal to another both by the feces and milk excreted by mice with amyloidosis. Thus, mouse AApoAII amyloidosis has been demonstrated to be a “transmissible disease”. In this study, to further characterize the transmissibility of amyloidosis, AApoAII amyloid fibrils were injected into transgenic *Apoa2^c^*Tg*^+/−^* and normal R1.P1-*Apoa2^c^* mice to induce AApoAII systemic amyloidosis. Two months later, AApoAII amyloid deposits were found in the skeletal muscles of amyloid-affected mice, primarily in the blood vessels and in the interstitial tissues surrounding muscle fibers. When amyloid fibrils extracted from the skeletal muscles were subjected to Western blot analysis, apoA-II was detected. Amyloid fibril fractions isolated from the muscles not only demonstrated the structure of amyloid fibrils but could also induce amyloidosis in young mice depending on its fibril conformation. These findings present a possible pathogenesis of amyloidosis: transmission of amyloid fibril conformation through muscle, and shed new light on the etiology involved in amyloid disorders.

## Author Summary

“Amyloidosis”, a group of protein folding diseases characterized by deposition of fine fibrils in tissues, is a common disorder of protein metabolism and can be acquired, inherited and/or age-associated. Recently, prion-like transmission has been found in various amyloidoses. AApoAII amyloid fibrils in mouse senile amyloidosis have exhibited transmissibility. For instance, ingested AApoAII amyloid fibrils, which were excreted from mice and contained in feces or milk, function as seeds for changing apoA-II amyloid precursor protein to the fibrillar form and cause mouse senile amyloidosis. However, transmissibility through other pathways has not yet been established. Here, we induced AApoAII systemic amyloidosis in transgenic *Apoa2^c^*Tg*^+/−^* and normal R1.P1-*Apoa2^c^* mice to analyze the transmissibility of mouse senile amyloidosis through muscle tissues. In this study, we not only detected AApoAII deposited in various skeletal muscles, but also found that it could induce secondary transmission of AApoAII amyloidosis. This is the first evidence of transmission through skeletal muscles in non-prion systemic amyloidosis. This pathway of transmission provides new insight into the potential for food-borne pathogenesis and etiology of systemic amyloidosis.

## Introduction

Amyloidosis refers to a group of protein folding disorders. Various proteins that are harmless and soluble under normal physiological conditions can undergo marked conformational changes and subsequent self-assembly outside of the cell into highly stable, insoluble amyloid fibrils with a high content of ß-sheet structures. Currently, twenty-eight different kinds of human proteins, in intact or fragmented forms, have been found to be amyloidogenic *in vivo* and to be associated with pathological disorders such as prion diseases, Alzheimer's disease, type II diabetes, dialysis-related amyloidosis, and familial, systemic, and sporadic amyloidosis [1], [2].

Many factors, such as aging and epigenetic factors, including lifestyle and types of food ingested, may influence fibril formation and deposition in organs and/or tissues. Transmission of amyloid fibrils might act as an important etiological factor of amyloidosis. Exogenous amyloid fibrils could act as a seed (nuclei) and change the conformation of endogenous amyloid protein into that of fibrils, such as PrP^Sc^, reactive (AA) and AApoAII amyloid fibrils [3], [4], [5]. Transmissible spongiform encephalopathies (TSEs) comprise a group of infectious neurodegenerative diseases that affect humans and other animals and are characterized by accumulation of the misfolded, protease-resistant prion protein PrP^Sc^ in the central nervous system [6], [7]. It is hypothesized that TSEs are transmitted from one species to another through ingestion of urine, saliva, and/or infected meat [7].

Apolipoprotein A-II (apoA-II) is present in the plasma of humans, mice, rats, and fish [8], [9]. In mice, apoA-II is the second most abundant apoprotein in serum high density lipoprotein (HDL), and accumulates to form amyloid fibrils (AApoAII) in many organs, leading to senile amyloidosis [10]. In laboratory mice, three major alleles (*Apoa2^a^*, *Apoa2^b^* and *Apoa2^c^*) of the apoA-II gene encode three variants of the apoA-II protein [11], [12]. Several genetic analyses have indicated that the *Apoa2^c^* allele markedly accelerates age-associated deposition of AApoAII [13]. Mouse AApoAII amyloidosis has been demonstrated to be a transmissible disease by a prion-like infectious process occurring through a seeding-nucleation mechanism [4], [14]. Our group found that a single intravenous injection of a very small amount of AApoAII amyloid fibrils (∼10*^−13^*g) led to systemic deposition of amyloid in young mice [15], [16]. AApoAII amyloidosis can also be transmitted by the feces [17] and milk [18] excreted by mice with AApoAII amyloidosis. Furthermore, transmission of AApoAII amyloidosis shows a ‘strain phenomenon’ analogous to the prion strains [10]. Thus, the fibrillar nuclei or amyloid fibrils formed by the aggregation of misfolded protein monomers (rich in ß-sheet structures) act as seeds to induce and stabilize conversion of the native monomeric protein [19], [20]. This mechanism provides a plausible explanation for the transmissible nature of AApoAII amyloidosis.

In the present study, we found AApoAII amyloid fibrils in the skeletal muscles of AApoAII amyloid-affected mice. Unexpectedly, amyloid fibrils isolated from the muscles were demonstrated to be sufficient for the transmission of amyloidosis. These findings provide important implications for assessing the potential risk of consuming amyloid-deposited skeletal muscles in the transmission of amyloidosis.

## Results

### Expression of apoA-II mRNA in muscle tissue

**Figure 1:**
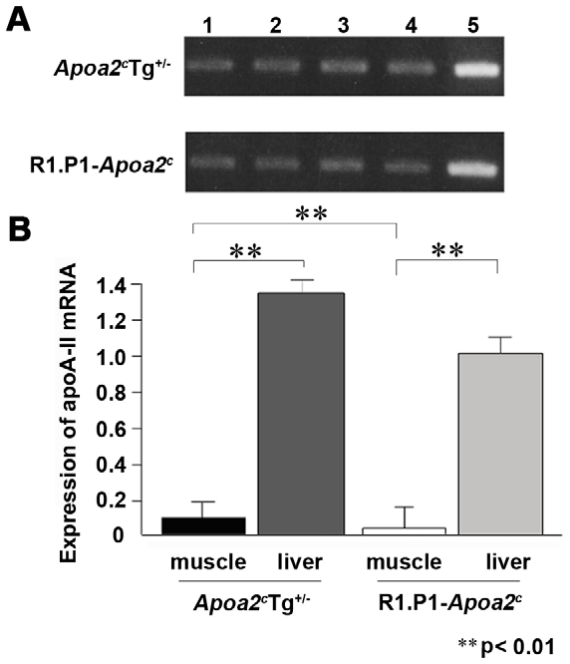
Expression of apoA-II mRNA in various skeletal muscles. A. Expression of apoA-II mRNA was determined by RT-PCR analysis with 23 cycles from four different muscles in *Apoa2^c^*Tg*^+/−^* and R1.P1-*Apoa2^c^* mice. Lane 1, forelimb triceps brachii muscles. Lane 2, hindlimb femoral quadriceps muscles. Lane 3, longissimus thoracis muscles from the back. Lane 4, greater pectoral muscles from the breast. Lane 5, liver as a control. B. Real-time PCR analysis of apoA-II mRNA levels in the greater pectoral muscle and the liver of the *Apoa2^c^*Tg*^+/−^* and R1.P1-*Apoa2^c^* mice (^**^p<0.01).

To confirm whether apoA-II mRNA is expressed in mouse skeletal muscle, total RNA was extracted from the triceps brachii muscles in the forelimbs, the femoral quadriceps muscles in the hindlimb, the longissimus thoracis muscle in the back and the greater pectoral muscles from the breast of *Apoa2^c^*Tg*^+/−^* mice and R1.P1-*Apoa2^c^* mice. ApoA-II mRNAs were detected in several muscles obtained from these mice. Interestingly, expression levels in muscle tissues were lower than those seen in liver (Figure 1A). Quantitative real time PCR analysis revealed that the expression levels of apoA-II mRNA in the muscles were about one tenth and one thirtieth of the expression levels observed in the livers from *Apoa2^c^*Tg*^+/−^* and R1.P1-*Apoa2^c^* mice, respectively, and expression levels were significantly higher in *Apoa2^c^*Tg*^+/−^* mice (Figure 1B).

### AApoAII amyloid deposits in the muscle

**Figure 2:**
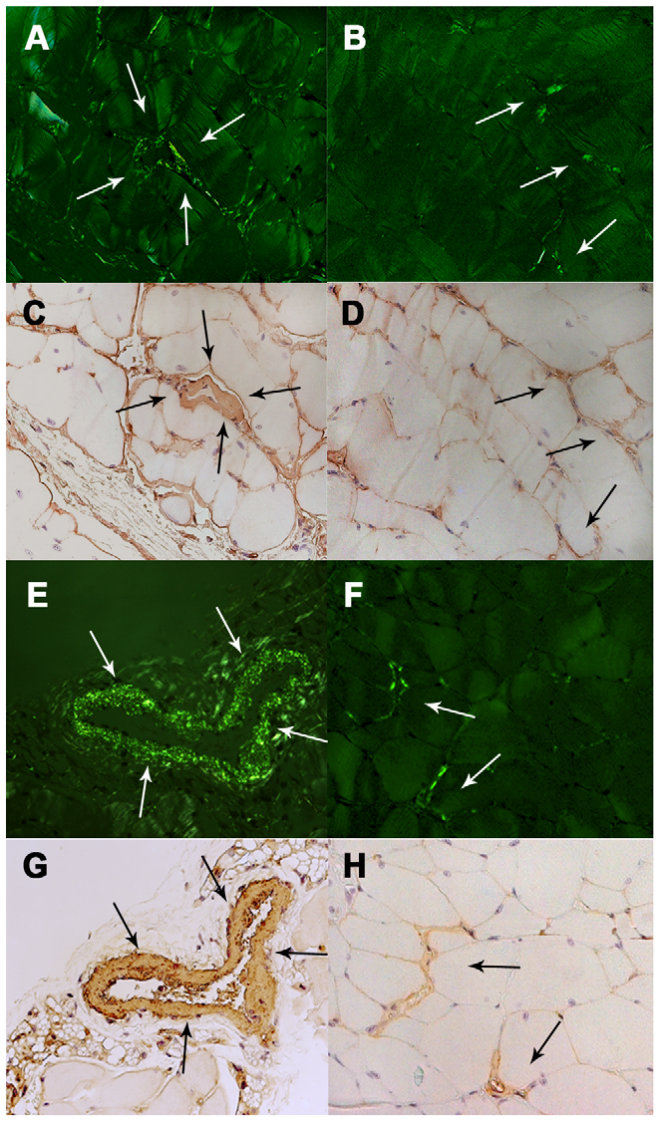
Amyloid deposition in the muscle. Amyloid deposits in the breast greater pectoral muscles from the 4-month-old *Apoa2^c^*Tg*^+/−^* mice two months after injection with AApoAII amyloid fibrils (A, B, C and D) and from 6-month-old R1.P1-*Apoa2^c^* mice with 4-month AApoAII amyloid induction (E, F, G and H). Amyloid depositions in the blood vessels (A and E) and interstitial tissues surrounding muscle fibrils (B and F) in the pectoral muscles manifest as green birefringence in Congo red-stained sections. Deposition of amyloid was identified with anti-apoA-II antiserum (C, D, G and H). The arrows indicate amyloid deposition in the muscle. Magnification = ×400.

To determine whether amyloid fibrils exist in the muscles of AApoAII-deposited mice, we intravenously injected 1 µg of isolated AApoAII fibrils into six 2-month-old female *Apoa2^c^*Tg*^+/−^*mice. Two months later, amyloid deposition was detected by the presence of green birefringence in Congo Red-stained tissue from four muscles of *Apoa2^c^*Tg*^+/−^* mice displaying heavy amyloid deposits throughout the body. Histological examination revealed that muscles of all *Apoa2^c^*Tg*^+/−^* were deposited with amyloid (6/6; Table 1). In amyloid fibril-injected normal R1.P1-*Apoa2^c^* mice, obvious amyloid deposition was observed only in one of three mice at two months after injection (1/3), and all three had amyloid deposits at four months after injection (3/3); that is, in skeletal muscles, the deposition of AApoAII amyloidosis increased with age. Amyloid deposits were found mainly in the blood vessels of muscle tissues, but were also found in connective tissues around muscle fibers (endomysium) both in *Apoa2^c^*Tg*^+/−^* mice (Figure 2A, B) and R1.P1-*Apoa2^c^* mice (Figure 2E, F). AApoAII amyloid deposition, which was observed in muscles, was further confirmed with anti-apoA-II staining (Figure 2C, D, G, and H). However, no deposits of AApoAII amyloid fibrils were found in muscle tissues of the R1.P1-*Apoa2^c^* mice (0/3) without induction by AApoAII fibrils.

**Table 1:** Deposition of AApoAII amyloid fibrils in the muscles of *Apoa2^c^*Tg*^+/−^* and R1.P1-*Apoa2^c^* mice.

Mouse strain	Injected fibrils (µg)	Time after injection with AApoAII fibrils (months)	Rate of amyloid deposition in the muscles (positive/total)*
*Apoa2^c^*Tg*^+/−^*	1	2	6/6
R1.P1-*Apoa2^c^*	100	2	1/3
R1.P1-*Apoa2^c^*	100	4	3/3
R1.P1-*Apoa2^c^*	0	2	0/3

AApoAII deposition was determined by staining with Congo red or anti-apoA-II antiserum in tissue sections taken from four muscles from each mouse.

*: number of mice with amyloid deposition in any of the four muscles.

**Figure 3:**
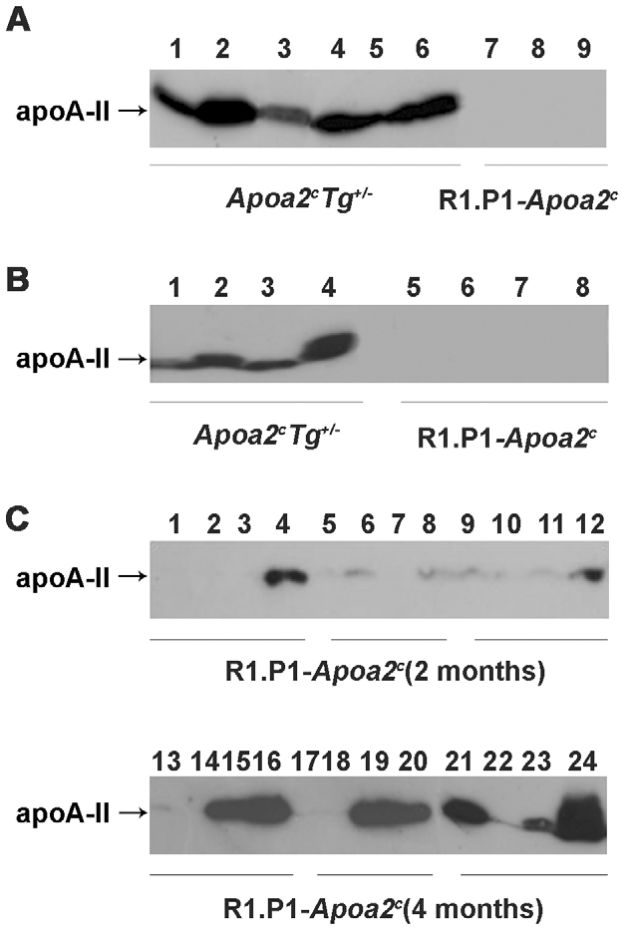
ApoA-II protein detected in amyloid fibril fractions isolated from various muscles. A. ApoA-II protein in amyloid fibril fractions of the hindlimb muscles from six *Apoa2^c^*Tg*^+/−^* mice injected with AApoAII (mouse-1 to mouse-6 in lane 1 to 6), and three R1.P1-*Apoa2^c^* mice without AApoAII injection (mouse-1 to mouse-3 in lane 7 to 9) were detected with anti-mouse apoA-II antiserum. 25 µg of protein were loaded into each lane. B. ApoA-II protein found in various muscles of *Apoa2^c^*Tg*^+/−^* mouse-1 injected with AApoAII (Lane 1 to 4) and a non-injected R1.P1-*Apoa2^c^* mouse-1 (lane 5 to 8). Lane 1 and 5: forelimb triceps brachii muscles. Lane 2 and 6: hindlimb femoral quadriceps. Lane 3 and 7: longissimus thoracis muscles in the back. Lane 4 and 8: greater pectoral muscles from the breast. C. ApoA-II protein found in various muscles of R1.P1-*Apoa2^c^* mice (two or four months after injection with AApoAII fibrils). Lane 1, 5, 9, 13, 17 and 21; forelimb triceps brachii muscles. Lane 2, 6, 10, 14, 18 and 22: hindlimb femoral quadriceps muscles. Lane 3, 7, 11, 15, 19 and 23: longissimus thoracis muscles in the back. Lane 4, 8, 12, 16, 20 and 24: greater pectoral muscles.

Amyloid fibril fractions were isolated from various muscles and apoA-II protein was detected by Western blot analysis. ApoA-II proteins were detected in amyloid fibril fractions of femoral quadriceps muscles in the hindlimb of *Apoa2^c^*Tg*^+/−^* mice with AApoAII-deposition but similar results were not observed in the muscles of R1.P1-*Apoa2^c^* control mice lacking AApoAII-deposition (Figure 3A). Moreover, apoA-II was also detected in all four kinds of muscle of *Apoa2^c^*Tg*^+/−^* mice (Figure 3B). In R1.P1-*Apoa2^c^* mice, two months after injection of amyloid fibrils, apoA-II was detected in greater pectoral muscles in the breast of all three mice. Four months after injection, apoA-II deposition expanded to other muscles and amounts of apoA-II increased (Figure 3C). The amount of deposition was different among different muscles: greater pectoral muscles from the breast > longissimus thoracis muscle in the back > triceps brachii muscles in the fore-limbs > femoral quadriceps muscles in the pelvic-limb.

### Detection of amyloid fibrils in muscle by transmission electron microscopy

**Figure 4:**
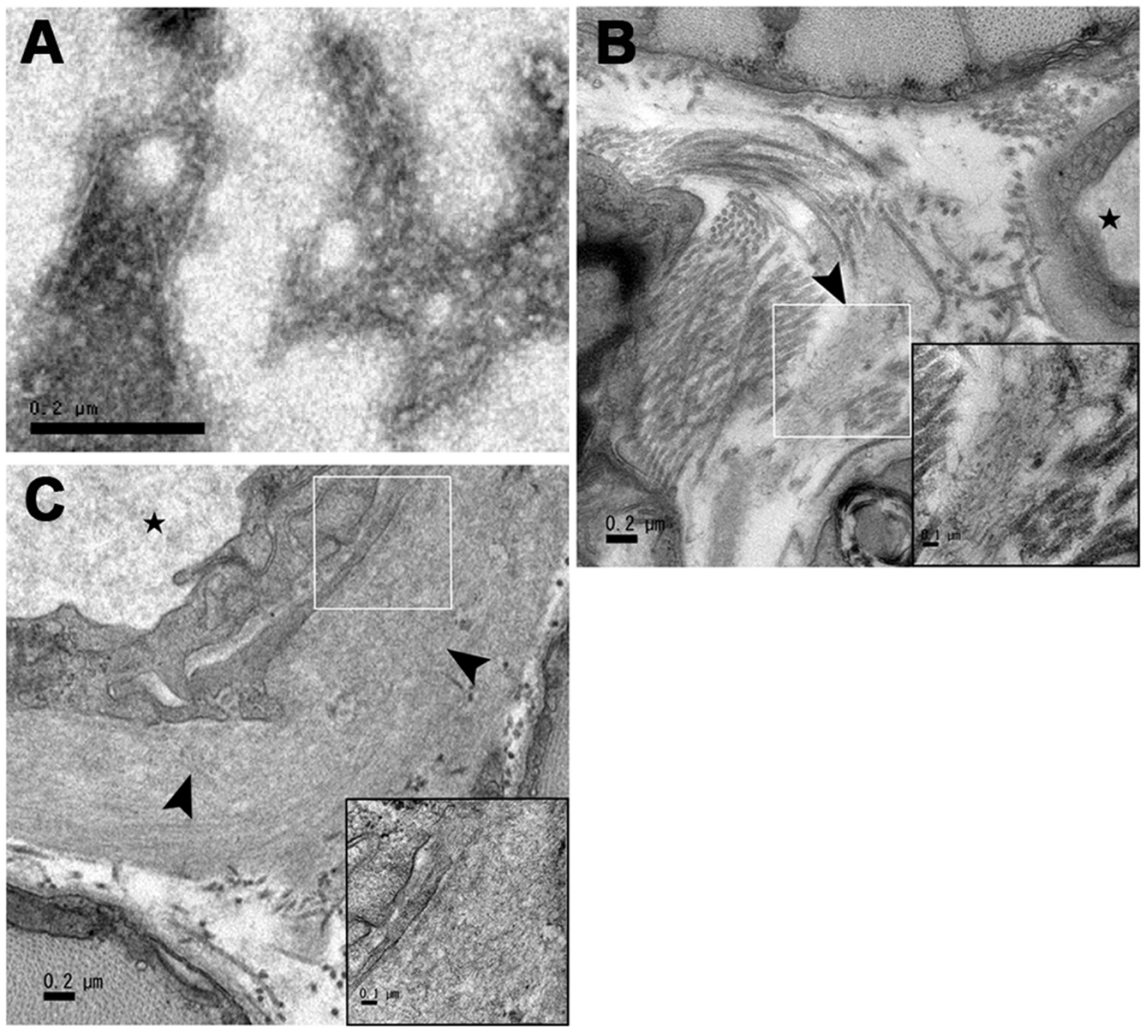
Transmission electron microscopy images of amyloid fibrils deposited in muscles. A. Amyloid fibrils extracted from the muscles of an AApoAII-deposited *Apoa2^c^*Tg*^+/−^* mouse corresponding to lane 1 in Figure 3A. Transmission of electron micrographs of pectoral muscles from the 4-month-old *Apoa2^c^*Tg*^+/−^* mice two months after injection and confirmed fibrillar deposits in the endomysium (4B) and capillary walls (4C). Arrowheads indicate the amyloid deposits. The highmagnification insets of the areas surrounded with white squares shows the fibrillar nature of the deposits. Scale bar = 0.2 µm.

To further confirm the existence of amyloid fibrils in muscle tissues, the amyloid fibril fractions of muscle tissues from the AApoAII-deposited *Apoa2^c^*Tg*^+/−^* mice and R1.P1-*Apoa2^c^* mice without AApoAII-deposition were observed by transmission electron microscopy. We found amyloid fibrils extracted from muscles only in the fractions of mice with AApoAII-deposition (Figure 4A), but not in the fractions of mice without deposition. Ultrastructural analysis of cross sections of AApoAII-deposited muscle was performed by transmission electron microscopy. Bundles of amyloid fibrils deposited in endomysiums and capillary walls of skeletal muscles were observed (Figure 4B and C).

### Induction of amyloidosis with amyloid fibril fractions isolated from AApoAII-deposited muscles

**Figure 5:**
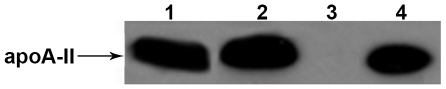
Western blot analysis of amyloid fibril fractions extracted from muscles used for secondary transmission. Lane 1, Amyloid fibril fractions (2.5 µg) isolated from femoral quadriceps muscles of *Apoa2^c^*Tg*^+/−^* mice with AApoAII deposition. Lane 2, Denatured muscle amyloid fibril fraction (2.5 µg) from *Apoa2^c^*Tg*^+/−^* mice. Lane 3, Muscle amyloid fibril fraction extracted (5.0 µg) from R1.P1-*Apoa2^c^* mice without amyloid injection. Lane 4, Amyloid fibril fraction from liver (1 µg) used as a control. ApoA-II was detected with anti-apoA-II antiserum.

To elucidate whether AApoAII amyloid transmissibility existed in skeletal muscle, amyloid fibril fractions were isolated from femoral quadriceps muscles of *Apoa2^c^*Tg*^+/−^* mice with AApoAII deposition (Figure 5) and were injected into 2-month-old female R1.P1-*Apoa2^c^* mice. Two months following injection, amyloid deposits were observed in the tongue (6/6), lungs (6/6), stomach (6/6), heart (6/6), intestine (4/6), and skin (1/6) of six injected mice (Table 2). The mean AI was 1.19. In contrast, significantly less amyloid deposition was observed when the mice were injected with fractions isolated from R1.P1-*Apoa2^c^* mice without AApoAII-deposition; the mean AI was 0.26 (p = 0.0062). Additionally, we found that injection of fibril fractions extracted from no AApoAII deposited muscles from R1.P1-*Apoa2^c^* mice induced a small amount of amyloid deposition (2/5 mice had AApoAII deposits and mean AI = 0.10). To confirm this, amyloid fibril fractions were extracted from muscles of young (2-month-old) female R1.P1-*Apoa2^c^* mice. Although these fractions induced secondary transmission in mice, neither apoA-II nor AA could be detected in the fraction by Western blot analysis (Figure 5). Unexpectedly, slight amyloid depositions were observed in the tongue (5/7) and stomach (3/7) of seven injected mice two months after injection; mean AI = 0.20.

**Table 2:** AApoAII amyloid deposits in R1.P1-*Apoa2^c^* mice injected with amyloid fibril fractions isolated from the muscles of AApoAII-deposited mice.

Injected fractions source	Positive/total	AI	p-value
Muscle of *Apoa2^c^*Tg*^+/−^* (with amyloid deposition)	6/6	1.19 *^a^*	p<0.01 (a vs b)
Muscle R1.P1-*Apoa2^c^* (without amyloid injection)	4/5	0.26 *^b^*	
Denatured amyloid fibril fraction (muscle of *Apoa2^c^*Tg*^+/−^*)	0/6	0 *^c^*	p<0.01 (a vs c)

Amyloid fibril fractions were isolated from femoral quadriceps muscles of *Apoa2^c^*Tg*^+/−^* mice with AApoAII deposition and were injected intravenously into 2-month-old female R1.P1-*Apoa2^c^* mice. After two months, mice were sacrificed and amyloid deposition and AI were determined.

### Denatured AApoAII could not induce the deposition of AApoAII amyloidosis

Amyloid fractions denatured by guanidine hydrochloride were injected into six 2-month-old female R1.P1-*Apoa2^c^* mice. No amyloid deposition was detected in any of these mice two months later (0/6) (Table 2).

## Discussion

Our previous work in senescence-accelerated mice determined that AApoAII amyloid fibrils are deposited throughout the body, including in the liver, spleen, stomach, intestine, heart, kidneys, lungs, tongue, skin, gonads, adrenal glands, salivary and thyroid glands, thymus, mesenteric lymph nodes, epineurium of the sciatic nerve and blood vessels in various tissues; however, no evidence of amyloid deposition was found in brain parenchyma or bone marrow in the vertebral body of the lumbar spine [21]. Although amyloid fibrils were also detected in the musculoskeletal systems, there were no detailed descriptions nor further studies on AApoAII amyloid deposition in the skeletal muscles [21].

The current study demonstrates that skeletal muscle tissue is capable of propagating AApoAII amyloidosis in mice. We first detected AApoAII amyloid fibrils in four skeletal muscles in different body regions using immunohistochemistry, Western blot analysis and electron microscopy. Amyloid deposits were observed in blood vessels and interstitial tissues surrounding muscle fibers. However, no AApoAII was observed in muscle cells or in the nerve fibers in which prion proteins were previously detected [22]. Although apoA-II mRNA was detected in muscle tissues (Figure 1), it is unclear whether apoA-II protein in AApoAII fibrils around muscle fibers originates from muscle cells or blood. Interestingly, the amounts of amyloid deposition in the skeletal muscles differed among body regions; that is, breast > back > forelimb > hindlimb. This order is different from that observed in mice inoculated with prion protein [23]. First, we observed amyloid deposition in *Apoa2^c^*Tg*^+/−^* mice in which apoA-II protein is overexpressed in various tissues under the control of a ubiquitous promoter, and found that serum levels of apoA-II increased the susceptibility to induction of AApoAII [24]. As a result, AApoAII deposits in muscles were found in *Apoa2^c^*Tg*^+/−^* mice. Second, we also observed AApoAII deposits in the muscles of R1.P1-*Apoa2^c^* mice in which apoA-II was expressed under an endogenous promoter/enhancer. According to the above results, it was found that AApoAII is deposited in the skeletal muscles as part of a universal phenomenon. Next, we demonstrated that intravenous injection of amyloid fibrils extracted from muscle tissues could transmit amyloidosis depending on fibril-conformation. That is, transmissibility was lost following denaturation with 6 mol/L guanidine hydrochloride. In previous studies, AApoAII amyloid fibril was extremely efficient in inducing amyloidosis following doses of less than 1 pg; moreover, amyloidosis could be initiated after oral ingestion of AApoAII fibrils [16], [17], [25]. Thus, the infectious ability of skeletal muscle raised the possibility that mouse AApoAII amyloidosis may result, in part, from dietary exposure to amyloid fibrils through consumption of muscle/meat containing amyloid materials.

Animal muscles, an important food component for most humans, have been examined in several studies for the presence of TSE transmissibility [23], [26]. Recently, it was reported that high prion titers and the disease-causing isoform of the prion protein PrP^Sc^ appear in the skeletal muscles of mice, hamsters and sheep inoculated with prion agents [27], [28], [29], [30], and in deer infected with chronic wasting disease [31]. Furthermore, PrP^Sc^ is also present in skeletal muscle samples of sporadic Creutzfeldt-Jacob disease (CJD) in humans [32], and has been demonstrated to be present in the nerve fibers of skeletal muscles tissue [23]. Although some findings are contradictory to the above reports [33], elucidation of the contribution of muscle tissues to transmission is important for the prevention of prion-related disorders. Prion-like transmission has been reported in mouse inflammation-associated amyloid A (AA) amyloidosis [34]. Dietary supply of amyloid fibrils might also be a trigger in the development of AA amyloidosis, especially for a susceptible population. Notably, it was reported that 71.4% of skeletal muscles from cows with systemic AA amyloidosis stained positive with anti-AA antibody [35]. Although an unexpectedly high incidence of visceral AA-amyloidosis in aged slaughtered cattle in Japan was reported, and isolated AA amyloid fibrils exhibited amyloid-enhancing factor activity, amyloid deposition in the skeletal muscles was rare [36], [37].

Thus, these studies support the idea of the transmissibility of systemic AApoAII and AA amyloidosis from skeletal muscle. Additionally, we found that injection of fibril fractions extracted from either control non-AApoAII injected or, no AApoAII deposited muscles of young R1.P1-*Apoa2^c^* mice induced a small amount of amyloid deposition, although these fractions contained neither apoA-II nor AA detectable by Western blot analysis. It is possible that extracts contain trace amounts of AApoAII amyloid fibrils or oligomers that could not be detected by available techniques and these undetectable AApoAII peptides might be transmissible like PrP^res^ [38]. Alternatively, components other than AApoAII amyloid fibrils might induce amyloid deposition. In R1.P1-*Apoa2^c^* mice, AApoAII amyloid can be seeded by various heterogeneous amyloid fibrils (cross-seeding) [39] and unexpectedly many kinds of proteins have been reported to form amyloid fibril-like structures [40], [41]. For example, collagen fibrils and glycosaminoglycans, supportive structures in skeletal muscle, appear to be actively involved in the induction of ß_2_-microglobulin amyloid fibril formation [42].

Elevated levels of prion protein in muscle lead to myopathy and neurogenic muscle atrophy in affected patients [43], [44], [45]. Accumulated amyloid proteins have been found in inclusion-body myositis and are toxic to skeletal myoblasts [46]. Although we observed amyloid deposits around skeletal muscle fibers after inducing amyloidosis, further studies will be necessary to examine possible myopathy and/or toxicity of these deposits.

In summary, apoA-II, which is present in the plasma of humans, mice, rats, and fish [6], [7], has been demonstrated in the form of amyloid fibrils with transmissibility in mouse muscle. AApoAII amyloid fibrils were detected in various skeletal muscles, especially in the pectoral muscles. The verification of this transmission pathway is valuable for understanding the pathogenesis and etiology of amyloidosis.

## Materials and Methods

### Ethics statement

All experimental procedures were pre-approved by Division of Laboratory Animal Research of Shinshu University and were performed according to the guidelines of Division of Laboratory Animal Research of Shinshu University.

### Experimental procedures

R1.P1-*Apoa2^c^* is a congenic strain of mice with the amyloidogenic *Apoa2^c^* allele from the SAMP1 strain in the genetic background of SAMR1 [13]. *Apoa2^c^* transgenic mice (*Apoa2^c^*Tg*^+/−^*) were established in the genetic background of R1.P1-*Apoa2^c^* [24]. These strains were maintained by sister-brother mating in the Division of Laboratory Animal Research, Research Center for Human and Environmental Science, Shinshu University. Mice were raised under specific pathogen-free (SPF) conditions at 24±2°C with a light-controlled regimen (12-hour light/dark cycle). A commercial diet (MF; Oriental Yeast, Tokyo, Japan) and tap water were provided *ad libitum*. In this study, only female mice were used to avoid AA amyloidosis and/or other adverse impacts caused by fighting or other behaviors among mice reared in the same cage. All experiment procedures were carried out in accordance with the Regulations for Animal Experimentation of Shinshu University.

### Detection of apoA-II mRNA expression in skeletal muscle

Total RNAs were extracted from the skeletal muscles and liver (as control) using RNeasy Mini Kit (Qiagen, Hilden, Germany) according to the manufacturer's instructions. First-strand cDNA was synthesized from 1 µg total RNA of each muscle tissue (First-strand cDNA Synthesis Kit; Amersham Pharmacia Biotech, Piscataway, NJ) and subjected to PCR amplification with *Taq* DNA polymerase (Promega; Madison, WI). The reverse transcriptase-polymerase chain reaction (RT-PCR) amplification was carried out in a 50-µl reaction mixture containing 200 µM each dNTP, 1× buffer containing 1.5 mM MgCl_2_, 0.1 µM each primer, and 1.25 U of *Taq* DNA polymerase. The cycling parameters for RT-PCR were initial denaturation for 1 minute at 94°C followed by 23 cycles of 30 sec at 94°C, 30 sec at 55°C, and 1 min at 72°C. A 5-µl aliquot of the PCR product was subjected to 3% agarose (Takara Bio, Otsu, Japan) gel electrophoresis. SYBR Premix Ex Taq II (Takara Bio) was used as the fluorescent marker to monitor DNA accumulation in quantitative real time PCR analysis with the 7500 Real-Time PCR System (Applied Biosystems Life Technologies, Tokyo Japan). The primers used for PCR amplification of apoA-II mRNA were as follows: A2/acc-F (5′-AAGAGACAGGCGGACGGACA-3′) and A2/acc-R (5′-GAGGTCTTGGCCTTCTCCAC-3′).

### Induction of AApoAII amyloidosis and preparation of animal tissue samples

The AApoAII amyloid fibril fraction was isolated as a water suspension from the livers of a 20-month-old R1.P1-*Apoa2^c^* mouse as described previously [10]. Purified amyloid fibrils were re-suspended at a concentration of 1.0 mg/ml in distilled water (DW). One milliliter of this solution was put into an Eppendorf tube and sonicated on ice for 30 sec with an ultrasonic homogenizer VP-5S (Tietech Co., Ltd., Tokyo, Japan) at power level 4. This procedure was repeated five times at 30 sec intervals. Sonicated amyloid samples were then immediately injected into the caudal vein of mice to induce AApoAII amyloidosis.

Six 2-month-old female *Apoa2^c^*Tg*^+/−^* mice were injected intravenously with 1 µg of AApoAII fibrils to induce AApoAII amyloidosis. Two months later the mice were sacrificed and the triceps brachii muscles in the forelimbs, the femoral quadriceps muscles in the hindlimb, the longissimus thoracis muscles in the back, and the greater pectoral muscles from the breast were dissected. Half of the tissue was kept at −80°C and the other half was fixed in 10% neutral buffered formalin, embedded in paraffin, and cut into 4-µm sections. Six 2-month-old female R1.P1-*Apoa2^c^* mice were injected intravenously with 100 µg of AApoAII fibrils; three were sacrificed after two months and the other three were sacrificed after four months. Muscle tissue was dissected and either stored or fixed and embedded in the same fashion as the *Apoa2^c^*Tg*^+/−^* transgenic mice. Three female R1.P1-*Apoa2^c^* littermates not injected with fibrils, were sacrificed at two months as controls.

### Isolation of AApoAII amyloid fibrils from muscle

AApoAII amyloid fibril fractions were isolated from the muscle of amyloid fibril-injected mice by Pras' method [47]. Thawed muscles (0.1 g) were sonicated twice for 30 sec with a 30-second rest interval in 1.0 ml of 0.15 M NaCl on ice using an ultrasonic homogenizer VP-5S (Tietech Co., LTD, Tokyo, Japan) at power level 4. The homogenate was centrifuged at 40,000×g for 20 min at 4.0°C, after which the supernatant was discarded and the pellet was re-suspended in 1.0 ml 0.15 M NaCl. The sonication and centrifugation were repeated two more times, and the pellet was suspended in 1.0 ml deionized DW (DDW) and centrifuged after sonication once more. The pellet was re-suspended with DDW and sonicated. Following centrifugation at 30,000×g for 20 min at 4.0°C, the supernatant containing amyloid fibrils was collected and used for Western blotting, transmission electron microscopy analysis and for the secondary transmission experiment.

### Detection of AApoAII fibrils in the muscles by histology and immunohistochemistry

Deposition of amyloid fibrils was identified by the appearance of green birefringence in Congo Red-stained sections [48] visualized under polarizing microscopy. AApoAII amyloid fibril proteins were identified immunohistochemically using the avidin-biotinylated horseradish peroxidase complex method with specific antiserum against mouse apoA-II (1∶3000) [49].

### Detection of AApoAII fibrils in muscle by Western blot examinations

Isolated amyloid fibril fractions (25 µg) from the muscles were separated on Tris-Tricine sodium dodecyl sulfate-polyacrylamide (16.5% [w/v] acrylamide) electrophoresis (SDS-PAGE) gels [50]. Proteins on the gel were electrophoretically transferred to Immuno-Blot polyvinylidene difluoride membrane (0.2 µm pore size; Bio-Rad, Hercules, CA, USA). Proteins on the membrane were detected with rabbit anti-mouse apoA-II antiserum (1∶3000), followed by peroxidase-conjugated goat IgG against rabbit immunoglobulin (1∶1000; ICN Pharmaceuticals, Inc., Aurora, OH, USA). Immunoreactive proteins were visualized with ECL reagents (Amersham Biosciences, Buckinghamshire, England). The film (Amersham Biosciences, Buckinghamshire, England) was exposed for 3 min.

### Detection of AApoAII fibrils in muscle tissue by transmission electron microscopy

Aliquots (20 µl; 0.5 µg/µl) of amyloid fibril fractions isolated from the muscles were applied to 400-mesh collodion-coated copper grid (Nissin EM Co., Ltd., Tokyo, Japan) for 1 min and subjected to negative staining with 1% phosphotungstic acid (pH 7.0) for 1 min. The negatively stained samples were observed with a JEOL 1200 EX electron microscope (JEOL, Tokyo, Japan) operated at 80 kV. Electron micrographs were taken with a Gatan multiscan camera model 791 with Gatan digital micrograph software version 3.6.4 (Gatan, Pleasanton, CA, USA). For ultrastructural analysis by electron microscopy, greater pectoral muscles were thinly sliced and placed in 2.5% glutaraldehyde at 4°C overnight. The tissue was rinsed twice with 0.1 M phosphate-buffered saline (PBS) and post-fixed with 1% osmium tetroxide on ice for 1 hour. Then, the tissue underwent a graded ethanol dehydration series and was infiltrated using a mixture of one-half propylene oxide and one-half resin for one hour. One hour later, the tissue was embedded in resin for four hours and then polymerized at 37°C for 10 hours followed by 60°C for 24 hours. One hundred nanometer sections were cut and stained with 4% uranyl acetate for 20 minutes and 0.5% lead citrate for five minutes. The sections were observed with a JEM-1400 transmission electron microscope (JEOL, Tokyo, Japan) operated at 80 kV. Electron micrographs were taken with a Gatan multiscan camera with Gatan digital micrograph software version 1.81.78 (Gatan, Pleasanton, CA, USA).

### Secondary transmission of amyloid fibrils isolated from muscle

Amyloid fibril fractions were isolated from the muscles of AApoAII-deposited *Apoa2^c^*Tg*^+/−^* mice or R1.P1-*Apoa2^c^* mice without AApoAII-deposition. 100 µl (2.5 µg/µl) of the amyloid fibril fractions were injected into two-month-old female R1.P1-*Apoa2^c^* mice, and after two months, the mice were sacrificed and the intensity of AApoAII amyloid deposition was determined semi-quantitatively using the amyloid index (AI). The AI was determined by taking the mean value of the scores of amyloid deposition (graded from 0 to 4) in the seven major organs (liver, spleen, tongue, heart, intestine, stomach, and skin) stained with Congo Red as described previously [10].

Amyloid fibril fractions were denatured in a solution of 6 mol/L guanidine hydrochloride, 0.1 mol/L Tris-HCl (pH 10.0), and 50 mmol/L dithiothreitol (1.0 mg/mL) for 24 hours at room temperature with gentle stirring. Denatured amyloid fractions were dialyzed quickly against 10 mmol/L NH_4_HCO_3_ solution. The solution was injected into two-month-old female R1.P1-*Apoa2^c^* mice that were treated as described above.

### Statistical analysis

We used the StatView software package (Abacus Concepts, Berkeley, CA, USA) to perform statistical analyses. Significant differences in the value of AI among the various groups of mice were examined using the Mann-Whitney *U*-test.
